# Novel mutation in the hepatocyte nuclear factor 1B/maturity – onset diabetes of the young type 5 gene – unreported Vietnamese case

**DOI:** 10.1186/1687-9856-2015-S1-P25

**Published:** 2015-04-28

**Authors:** Vu Chi Dung, Bui Phuong Thao, Can Thi Bich Ngoc, Nguyen Ngoc Khanh, Nguyen Phu Dat, Sian Ellard, Maria Craig

**Affiliations:** 1 Department of Endocrinology, Metabolism and Genetics, National Hospital of Pediatrics, Hanoi, Vietnam; 2 Department of Molecular Genetics, Royal Devon & Exeter NHS Hospital. Peninsula Medical School. UK; 3 School of Women's and Children's Health, UNSW Medicine, Sydney, NSW Australia

## 

Maturity-onset diabetes of the young type 5 (MODY5), a type of dominantly inherited diabetes mellitus and nephropathy, has been associated with mutations of the hepatocyte nuclear factor-1 (HNF-1β) gene, mostly generating truncated protein. Various phenotypes are related to HNF-1β mutations. Our aim to describe clinical and genetic findings in the unreported Vietnamese case identified with HNF-1β mutations. The proband with kidney failure from 7.5 years of age and diabetes diagnosed at 13.5 years of age who were described. Case report included information: characteristics of diabetes, renal function and structure, pancreas structure. Genomic DNA were extracted from WBC of whole blood and HNF-1β mutation was performed using PCR and direct sequencing. The proband is heterozygous for a novel HNF1B missense mutation (c.505T>C; p.Y169H). This mutation results in the substitution of the amino acid histidine (charged polar) for tyrosine (uncharged polar) at codon 169**.** The tyrosine residue is conserved across species and it is therefore likely that the p.Y169H mutation is pathogenic. This result is consistent with a diagnosis of renal cysts and diabetes syndrome (RCAD). Testing was done for proband's parents and no mutation was found in HNF1B. It is therefore likely that the p.Y169H mutation has arisen de novo. Kidney MRI showed right kidney atrophy and pancreas MRI showed only tissue of head of pancreas. Investigations at 14.5 years of age – diagnosed diabetes showed: plasma urea 10.1 mmol/l; creatinine 250 micrommol/l; HbA1C 13.6%. He was given insulin of 0.8 UI/Kg/day and HbA1C was 6.8% after one year of treatment with insulin injection. Maturity-onset diabetes of the young type 5 encompasses a wide clinical spectrum. Analysis for mutations of HNF-1β is warranted, even without a family history of diabetes, in non obese patients with diabetes and slowly progressive non diabetic nephropathy, particularly when pancreatic atrophy.

Written informed consent was obtained from the patient for publication of this Case report (and any accompanying images). A copy of the written consent is available for review by the Editor-in-Chief of this journal.

